# Antimutagenic components in *Spatholobus suberectus* Dunn against *N*-methyl-*N*-nitrosourea

**DOI:** 10.1186/s41021-019-0137-4

**Published:** 2019-12-11

**Authors:** Keiko Inami, Yoshihisa Asada, Takumi Harada, Yuta Okayama, Noriko Usui, Masataka Mochizuki

**Affiliations:** 1 Faculty of Pharmaceutical Sciences, Sanyo-onoda City University, Daigakudo-ri 1-1-1, Sanyo-onoda-shi, Yamaguchi, 756-0884 Japan; 2 Faculty of Pharmaceutical Sciences, Tokyo University of Science, 2641 Noda-shi, Chiba, 278-8510 Japan

**Keywords:** Antimutagenicity, Formononetin, Genistein, Isoliquiritigenin, Medicarpin, Naringenin, Hydroxyl radical

## Abstract

**Background:**

An extract from *Spatholobus suberectus* (*S. suberectus*) Dunn has been reported to show potent antimutagenic effects against *N*-alkyl-*N*-nitrosoureas in *umu* screening. The aim of this study was to identify the antimutagenic components from extracts of *S. suberectus* against *N*-methyl-*N*-nitrosourea (MNU) in the Ames assay with *Salmonella typhimurium* strain TA1535 and to elucidate the antimutagenic mechanism of the flavonoids.

**Results:**

From the ethyl acetate fraction obtained from fractionation of the methanol extract of *S. suberectus* Dunn, medicarpin, formononetin and isoliquiritigenin were successfully isolated through a combination of normal- and reversed-phase chromatography. Genistein and naringenin, which were already reported to be contained in *S. suberectus* Dunn, were also tested for their antimutagenicity towards MNU, along with formononetin, isoliquiritigenin and medicarpin. Our results demonstrated that genistein, isoliquiritigenin, medicarpin and naringenin were antimutagenic against MNU without showing cytotoxicity. MNU is reported to cause not only DNA alkylation but also induce reactive oxygen species. The hydroxyl radical scavenging capacity of the flavonoids was correlated with the antimutagenic capacity, indicating that the hydroxyl radical scavenging activity was involved in their antimutagenicity towards MNU.

**Conclusions:**

It is important to prevent DNA damage by *N*-nitrosamines for cancer chemoprevention. Genistein, isoliquiritigenin, medicarpin and naringenin were demonstrated to possess an antigenotoxic effects against carcinogenic MNU due to their radical scavenging activity.

## Introduction

*N*-Nitroso compounds, are implicated as carcinogens in the human environment such as food, tobacco smoke, air, water and cosmetics [1–6]. In addition, *N*-nitroso compounds have been reported to be formed endogenously, mainly in the stomach and bowel, or in an infection site [2]. The endogenous formation of *N*-nitrosamines is a possible explanation for the epidemiological connection of gastrointestinal cancers [7, 8]. Therefore, for chemoprevention, it is important to discover naturally occurring or synthetic compounds that can prevent the mutagenicity and carcinogenicity of *N*-nitrosamines.

*N*-Methyl-*N*-nitrosourea (MNU) is a direct-acting mutagen that forms the corresponding methyldiazohydroxides and methylates DNA to form DNA adducts that miscode during DNA replication, causing mutations that lead to tumour formation. MNU is formed by the nitrosation of methylurea with nitrite in guinea-pig stomachs, and MNU is absorbed from the stomach into the blood [9]. Additionally, MNU can be detected by the nitrosation of creatinine or fermented foods at gastric pH [10–12]. Therefore, for cancer chemoprevention, it is important to find some compounds that can inhibit the mutagenicity induced by MNU.

*Spatholobus suberect* (*S. suberectus*) Dunn (Leguminosae) is a traditional Chinese herbal medicine used to treat rheumatism, anaemia, menoxenia, and other disorders [13]. Pharmacologically, it exhibits anti-inflammatory [14, 15] and antitumour activities [16, 17]. Our previous study showed that an aqueous extract of *S. suberectus* Dunn showed the most potent antimutagenic effects against MNU by *umu* test screening [18].

The *umu* test is a convenient method because the data are obtained in a short period of time; however, the Ames test is a more precise method to evaluate the genotoxic activities of a wide variety of environmental carcinogens and mutagens than the *umu* test [19, 20]. The Ames test using bacterial strains of *Salmonella typhimurium* (*S. typhimurium*) is widely used by regulatory agencies, academic institutions and pharmaceutical companies to assess the mutagenic potential of compounds [21, 22]. In the present study, we identified antimutagens against MNU using the Ames assay, and the inhibitory mechanism was investigated.

## Materials and methods

### General experimental procedures

The reaction progress was monitored using thin-layer chromatography (TLC) on silica gel 60 F_254_ (0.25 mm, Merck, Darmstadt, Germany). Column chromatography was performed using silica gel 60 (0.04–0.063 mm, Merck). Melting points were determined using a Yanaco (Tokyo, Japan) micro-melting point apparatus without correction. The LC system used was equipped with an LC-6 AD pump (Shimadzu, Kyoto, Japan), a UVDEC-100 V spectrometric detector (JASCO, Tokyo, Japan), a YRD-880 IR detector (Shimamura Tech. Co. Ltd., Tokyo, Japan), or a SPD-20A (Shimadzu), and a Capcell pack RP-18 column (Shiseido, Tokyo, Japan). NMR spectra were recorded with a JEOL JNM-LA400 spectrometer (Tokyo, Japan). The chemical shifts were expressed as ppm downfield from TMS. The mass spectra were measured on a JEOL JMS-SX102A mass spectrometer. The ESR spectra were collected on a JEOL JES-X320.

### Reagents

Bacto agar and Bacto nutrient broth were obtained from Becton Dickinson Microbiology Systems (Sparks, USA). 5,5-Dimethyl-1-pyrroline *N*-oxide (DMPO) and ethylenediamine-*N,N,N′,N′*-tetraacetic acid disodium salt dihydrate (EDTA) were purchased from Dojindo Laboratories (Kumamoto, Japan). Sodium ammonium hydrogen phosphate tetrahydrate was obtained from Merck (Darmstadt, Germany). *S. suberectus* Dunn was purchased from Matsumoto Yakugyo Co., Ltd. (Nagoya, Japan). Genistein [mp. 304 °C (decomp.), recrystallized from ethanol and H_2_O] and isoliquiritigenin [mp. 197 °C, recrystallized from ethanol and chloroform] were obtained from Tokyo Kasei Kogyo Co., Ltd. (Tokyo, Japan). Formononetin (mp. 256 °C) and naringenin (mp. 251 °C) was purchased from LKT Laboratories, Inc. (Minnesota, USA). All reagents used were of the best commercially available quality from Wako Pure Chemical Industries (Osaka, Japan) and were not further purified unless otherwise noted. Medicarpin (mp. 194 °C) was synthesized according to the method of Goel et al [23]. MNU [mp. 106 °C (decomp.) was prepared as described previously [24]. Purity of the synthesized medicarpin and MNU were determined by the ^1^H-NMR spectra, which all required integral values matches the number of protons without any other peak.

### Preparation of the aqueous extract of *S. suberectus* Dunn

Dried *S. suberectus* Dunn was cut with a pair of scissors. Distilled water (130 mL) was added to the cut *S. suberectus* Dunn (30 g) and refluxed for 30 min. The mixture was filtered with suction, and the filtrate was concentrated under reduced pressure. The whole procedure was repeated twice. The crude extracts were combined, dried under vacuum, and finally gave a reddish solid (5.30 g).

### Preparation of the methanol extract of *S. suberectus* Dunn

Dried *S. suberectus* Dunn was cut with a pair of scissors. Methanol (130 mL) was added to the cut *S. suberectus* Dunn (30 g) and stirred for 30 min at room temperature. The mixture was filtered with suction, and the filtrate was concentrated under reduced pressure. The whole procedure was repeated twice. The crude extracts were combined, dried under vacuum, and finally a reddish solid (2.32 g) was obtained.

### Fractionation of the methanol extract of *S. suberectus* Dunn based on the solubility in organic solvents

Methanol (3.5 L) was added to the cut *S. suberectus* Dunn (1 kg) and incubated overnight at room temperature. The mixture was filtered with suction, and the filtrate was concentrated under reduced pressure. The whole procedure was repeated 5 times. The crude extracts were combined, dried under vacuum, and finally a reddish solid (162 g) was obtained. Ethyl acetate (300 mL) and water (300 mL) were added to the solid, and the aqueous phase was separated. The aqueous phase was re-extracted with ethyl acetate (150 mL × 6), and the combined organic phases were washed with water (100 mL). The aqueous phase was extracted with water-saturated *n*-butanol (150 mL × 6), and the combined organic phases were washed with *n*-butanol-saturated water (100 mL). The ethyl acetate, *n*-butanol and aqueous extracts were filtered, and the filtrates were removed of solvent with a rotary evaporator, and the residue was dried in vacuo. Finally, the ethyl acetate fraction (23.8 g), *n*-butanol fraction (48.0 g), and aqueous fraction (86.0 g) were obtained from the methanol extract of *S. suberectus* Dunn. The recovery of the weight was 97%.

### Isolation of antimutagenic compounds from the ethyl acetate fraction

From the ethyl acetate fraction obtained from the methanol extract of *S. suberectus* Dunn, medicarpin, formononetin and isoliquiritigenin were successfully isolated through a combination of normal and reversed systems of chromatography (see the additional file 1). The three purified compounds were characterized by comparing their spectroscopic properties with literature values.

### Bacterial mutation assay

The antimutagenic effect of each plant extract was assayed according to the Ames method using the plate-incorporation protocol [21, 22]. Dr. T. Nohmi (National Institute of Health Sciences, Tokyo, Japan) kindly provided the *S. typhimurium* TA1535.

A solution of MNU (1.5 μmol/50 μL of DMSO) was added to a test tube and supplemented with 0.1 M sodium phosphate buffer (pH 7.4, 0.5 mL), a solution (50 μL) with various concentrations of each fraction, and a culture of *S. typhimurium* TA1535 (0.1 mL), and the solution was thoroughly mixed. Then, top agar (2 mL) was added, and the mixture was poured onto a minimal-glucose agar plate. The revertant colonies were counted after incubation at 37 °C for 44 h. Experiments were performed in triplicate and repeated three times with similar results. The results are expressed as the mean ± SE. Plates with neither MNU nor plant extract were considered negative controls. MNU (1.5 μmol/50 μL) resulted in 1826 ± 52 colonies. All of the tested plates were microscopically examined for thinning, the absence of a background lawn and/or the presence of microcolonies, which are considered indicators of toxicity induced by the test material. Neither MNU nor the plant extracts displayed toxicity to *S. typhimurium* TA1535 under the conditions of the antimutagenicity test.

Mutagenic activity in the presence of the extracts is expressed as the percent of mutagenicity (% = Rs/R × 100), where Rs is the number of his^+^ revertants/plate for plates exposed to MNU and plant extracts, and R is the number of his^+^ revertants/plate of MNU. The number of spontaneous revertants was subtracted beforehand to give Rs and R. Thus, the mutagenicity of MNU in the absence of plant extracts was defined as 100% MNU mutagenicity.

### Cytotoxicity test

Toxicity assays under the same conditions as those used for the Ames test were performed to determine the maximum concentrations of each plant extract that could be added without exerting toxic effects on the bacteria used in the Ames test. A solution of MNU (1.5 μmol/50 μL of DMSO) was added to a test tube and supplemented with 0.1 M sodium phosphate buffer (pH 7.4, 0.5 mL), each solution of plant extract (50 μL), and a culture of *S. typhimurium* TA1535 (0.1 mL). A portion of the mixture was diluted 10^5^-fold times in 1/15 M PB. The diluted solution (200 μL) was supplemented with histidine-free top agar (2 mL) and poured on a nutrient broth agar plate. The colonies were counted after incubation at 37 °C for 20 h. Experiments were performed in triplicate and repeated three times with similar results. The results are expressed as the mean ± SE. A substance was considered cytotoxic when the bacterial survival was less than 80% of that observed in the negative control [25]. The mutation frequency was estimated as the number of mutants per 10^7^ surviving bacterial cells.

### Reaction with MNU and isoliquiritigenin

A solution of MNU (1.5 μmol/50 μL of DMSO) was added to a test tube and supplemented with 0.1 M sodium phosphate buffer (pH 7.4, 0.6 mL) and a solution of isoliquiritigenin (0.5 mg/50 μL of DMSO). DMSO (50 μL) was used instead of the isoliquiritigenin solution for the blank. The reaction conditions were similar to those of the mutation assay, although 0.1 M sodium phosphate buffer (pH 7.4, 0.1 mL) was used instead of a bacterial culture (0.1 mL). A portion of the mixture was diluted 10-fold in 0.1 M sodium phosphate buffer (pH 7.4) at specified intervals and the solution (1 μL) was injected into HPLC.

The kinetics of the reactions were followed by monitoring of the MNU peak by HPLC. A plot of [MNU] versus time gave a straight line with a slope of –*k*. The half-life was calculated from ln2/*k*. Experiments were repeated four times with similar results. The results were expressed as the mean ± SD.

MNU was determined using HPLC with a Shiseido capcell pack UG80 (5 μm, 250 × 4.6 mm) column with 8% methanol–H_2_O as the eluent at 0.7 mL/min and 254 nm.

Isoliquiritigenin was determined using HPLC with a Shiseido capcell pack UG80 (5 μm, 250 × 4.6 mm) column with 60% methanol–H_2_O as the eluent at 0.7 mL/min and 323 nm.

### Detection of DMPO-OH adducts by ESR spectroscopy

Each flavonoid was diluted in acetonitrile. To a test tube containing 0.9 M DMPO (10 μL, final conc. 40.9 mM), flavonoid (each concentration in 33 μL), 0.1 M sodium phosphate buffer (pH 7.4, 147 μL), 20 mM EDTA (10 μL, final conc. 0.91 mM) and 10 mM FeSO_4_ (10 μL, final conc. 0.45 mM) was added 10 mM H_2_O_2_ (10 μL, final conc. 0.45 mM), followed by mixing on a vortex mixer and then transferring to an aqueous flat-cell. Acetonitrile (33 μL) was used instead of compound solution for the blank. Further increasing the dose of flavonoid resulted in precipitation in the reaction mixture.

After 2 min, the ESR spectrum was acquired using the following parameters: magnetic field of 336.0 ± 5.0 mT, microwave power of 1.0 mW, modulation frequency of 9.42 GHz, modulation width of 0.05 mT, sweep time of 30 s, response time of 0.03 s, and receiver gain of 250. Experiments were repeated three times with similar results. The results were expressed as the mean ± SD.

The capacity of the ^•^OH scavenging activity at each flavonoid concentration was presented as a relative intensity determined by calculating the peak height of the ESR signal due to the ^•^OH adduct of DMPO (DMPO–OH). The ^•^OH scavenging activity in the presence of flavonoids is expressed as the percent of ^•^OH scavenging activity [% = (R-Rs)/R × 100], where Rs is the DMPO-OH adduct intensity in the presence of flavonoid and R is the DMPO-OH adduct intensity in the absence of flavonoid.

## Results and discussion

### Identification of the main component from the antimutagenic fractions

**Fig. 1:**
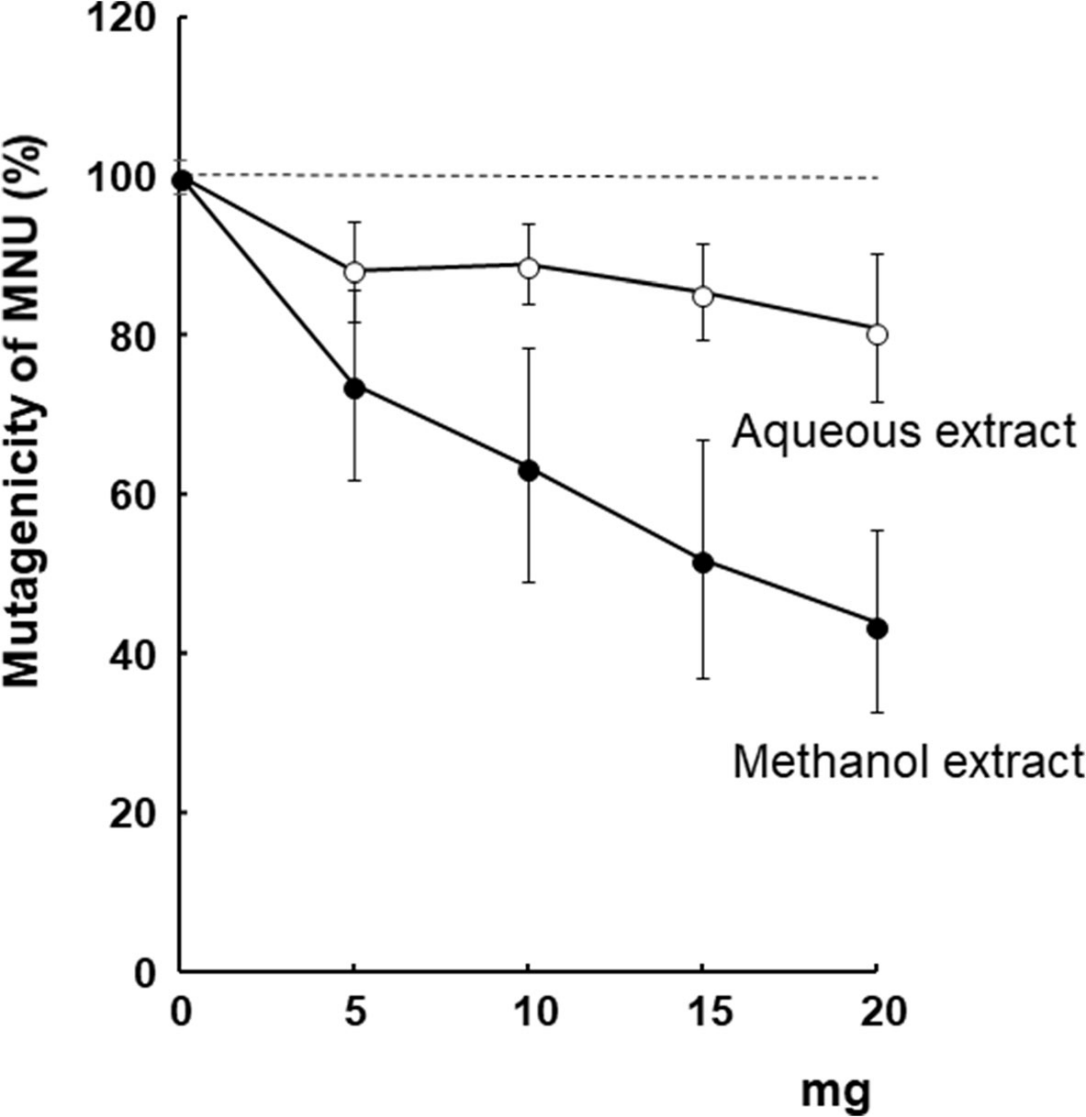
Effect of the extracts of *S. suberectus Dunn* on MNU-induced mutagenicity in *S. typhimurium* TA1535

The aqueous fraction of *S. suberectus* Dunn inhibited MNU-induced mutagenicity in preliminary screening using the *umu* test. To identify the antimutagenic component, the antimutagenic activity against MNU was compared among aqueous and methanol extracts (Fig. 1). The aqueous and methanol extraction yields were 10.6 and 4.6%, respectively. The methanol extract had a lower extraction yield; however, the antimutagenic activity in the methanol extract (40%) was better than that in the aqueous extract (25%) at a dose of 20 mg/plate on *S. typhimurium* TA1535.

**Fig. 2:**
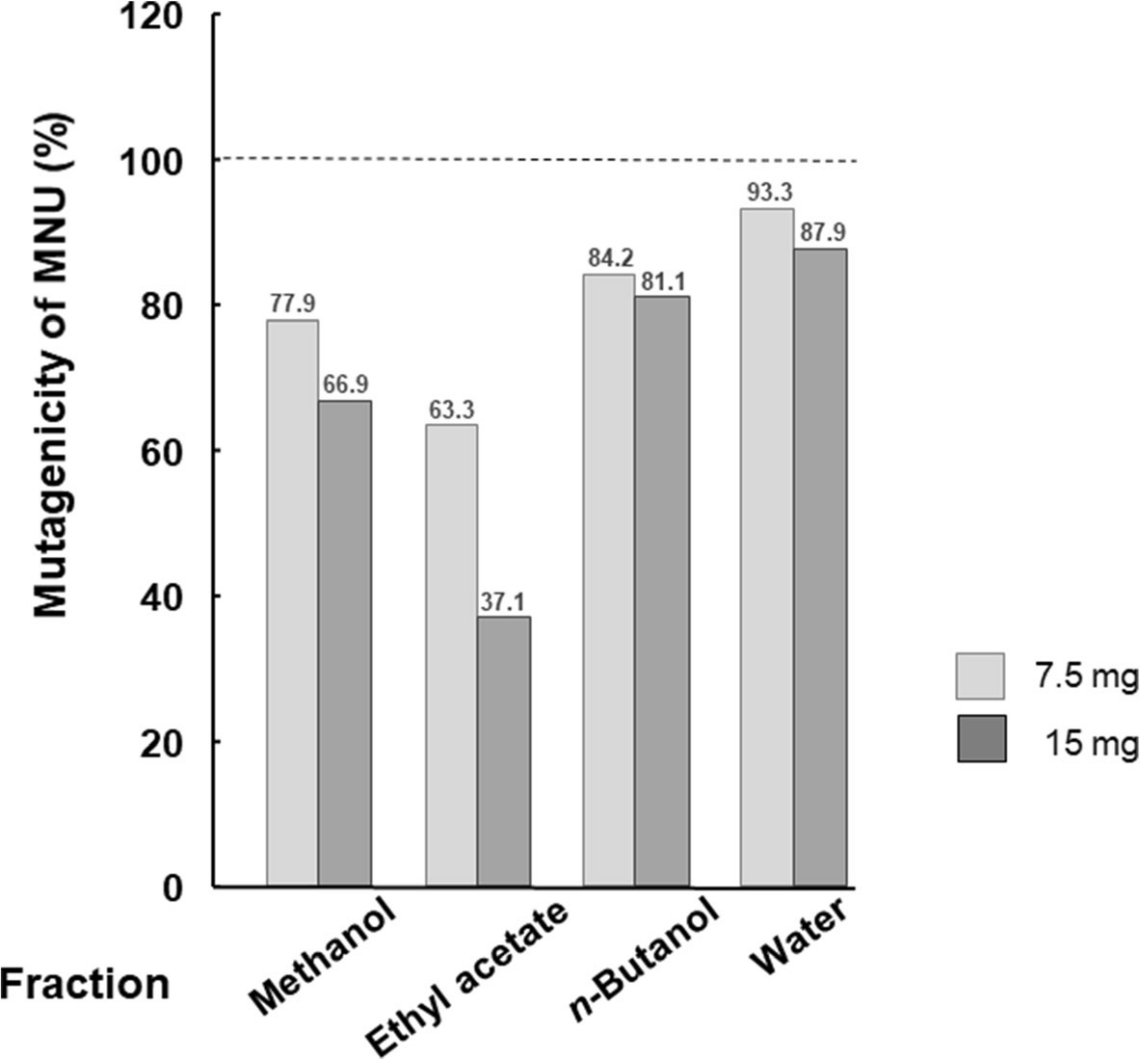
Effect of the fractions from the methanolic extracts of *S. suberectus* Dunn on MNU-induced mutagenicity in *S. typhimurium* TA1535

**Fig. 3:**
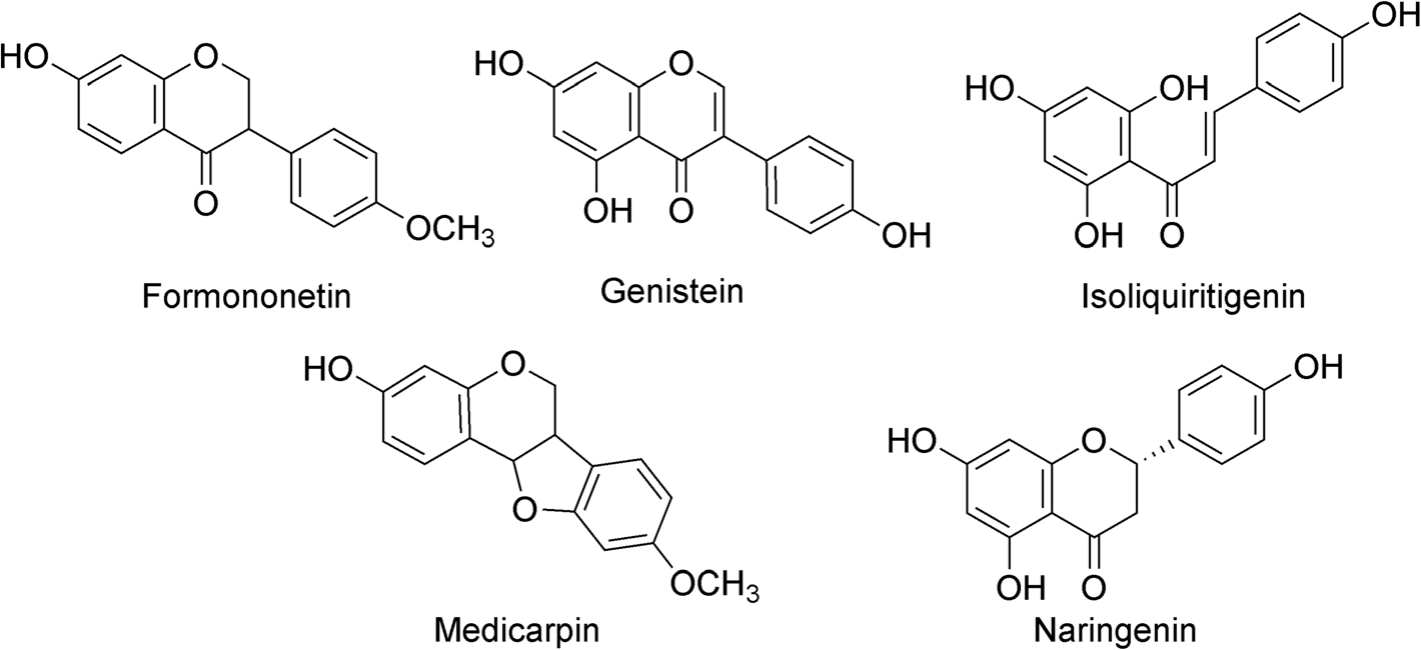
Structures of flavonoids from *S. suberectus* Dunn

The methanol fraction was sequentially partitioned into ethyl acetate, *n*-butanol and the residuals. Among these fractions, the ethyl acetate fraction (60% inhibition) showed the highest antimutagenic activity against MNU at a dose of 15 mg/plate in *S. typhimurium* TA1535 (Fig. 2). Therefore, the ethyl acetate fraction was separated through a combination of normal and reversed phases of chromatography (Fig. 3, additional file 1). In each fractionation step, the recoveries of the weights were more than 85% (see the additional file 1).

The methanol extract and its ethyl acetate fraction have appropriate polarity to yield a flavonoid-rich fraction [26]. In our study, the ethyl acetate fraction was prepared by partitioning the methanol extract of *S. suberectus* Dunn, which possessed the highest antimutagenicity, assuming that the antimutagens were flavonoids. Finally, three flavonoids were isolated and identified as formononetin, isoliquiritigenin and medicarpin, and these flavonoids have been reported to be isolated from *S. suberectus* Dunn [27, 28].

### Inhibitory effect of the components of *S. suberectus* Dunn on MNU-induced mutagenicity

Formononetin, isoliquiritigenin, and medicarpin were evaluated for their ability to inhibit MNU-induced mutagenicity in *S. typhimurium* TA1535 (Fig. 3). Additionally, genistein and naringenin were tested for their antimutagenicity towards MNU (Fig. 3). Genistein and naringenin have already been isolated from *S. suberectus* Dunn and are commercially available [29, 30].

**Fig. 4:**
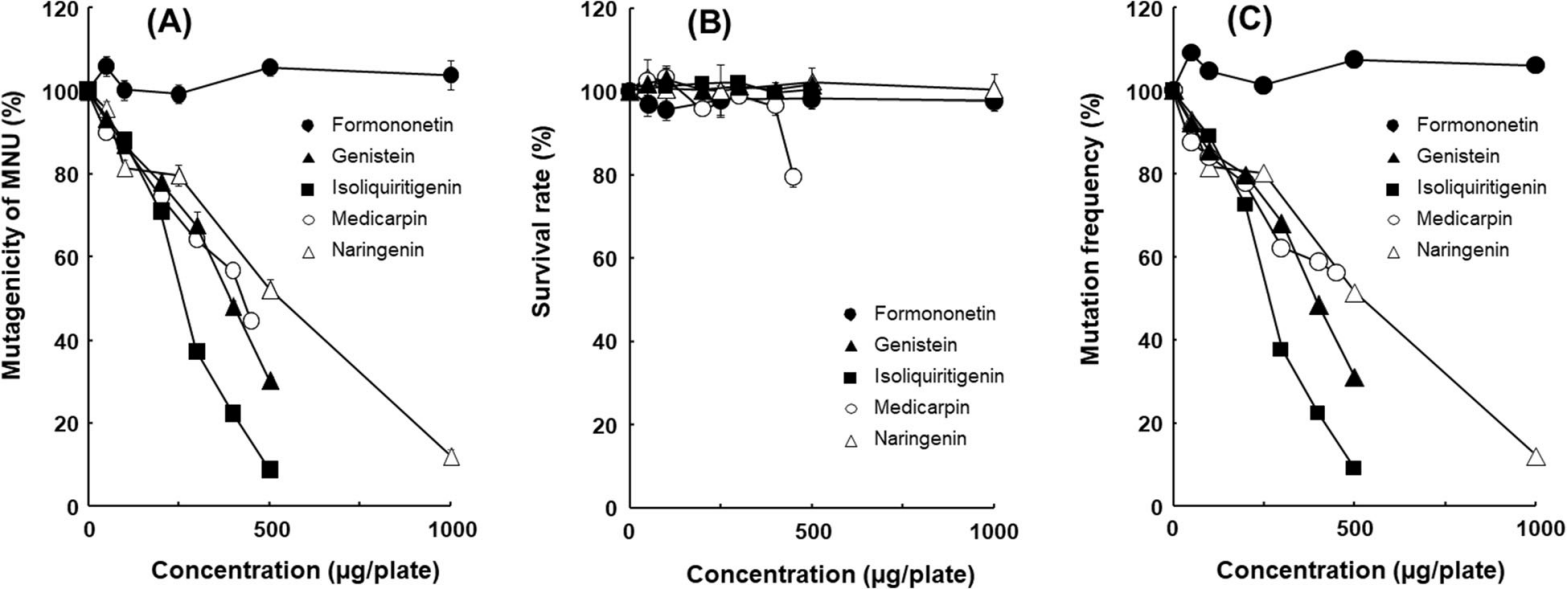
Mutagenicity (A), survival rate (B), and mutation frequency (C) of flavonoids on MNU-induced mutagenicity in *S. typhimurium* TA1535. The SE bars were too small to show

In the cytotoxicity assays, samples with values of > 80% viable cells were considered non-toxic compared with the viability of the negative control [25]. In this study, no cell toxic effects were observed at a concentration of 1.0 mg/plate for formononetin and naringenin and 0.5 mg/plate for genistein, isoliquiritigenin and medicarpin. To assess the precise antimutagenic potency of the plant extracts, the mutation frequency mutagenicity was calculated by dividing the number of mutants with the surviving fraction of bacteria. These data clearly showed that genistein, isoliquiritigenin, medicarpin and naringenin possessed antimutagenic activity against MNU in *S. typhimurium* TA1535 (Fig. 4). The antimutagenic activity of the flavonoids was of the following order: isoliquiritigenin > genistein > medicarpin = naringenin. Formononetin did not show antimutagenicity against MNU.

Flavonoids are well-known antimutagens that have been detected by Ames assays [31, 32], and there are several reports that use MNU as a mutagen [33]. Although genistein has been reported to inhibit MNU-induced mutagenicity, we also tested for its antimutagenicity towards MNU to compare with those of the other isolated flavonoids [34]. Naringenin has been reported to have antimutagenic activity against the indirect-mutagen aflatoxin B_1_ through inhibition of metabolic activating enzymes, but it did not show antimutagenicity against the direct-acting *N*-methyl-*N*′-nitro-*N*-nitrosoguanidine [35]. In this study, naringenin inhibited MNU-induced mutagenicity, which is the reason why the higher concentration of naringenin was used.

For the first time, isoliquiritigenin, naringenin and medicarpin were demonstrated to possess antimutagenic activity against MNU. These flavonoids have been reported to have anticancer activity [36–39], and the antimutagenic activity was considered to contribute to their anticancer activity.

### Hydroxyl radical-scavenging activity of the flavonoids derived from *S. suberectus* Dunn

The antimutagenic mechanism of the flavonoids in the *Salmonella* assay was reported to be by inhibition of enzymatic activation [40], induction of the SOS response [41], and the reaction between flavonoids and the metabolic antimutagenic-activating mutagen [42]. In this study, the direct-acting MNU was used, and the activity was evaluated using *S. typhimurium* TA1535, which did not contain the plasmid pKM101 [43]. Therefore, the inhibitory effect on the mutagenicity of direct-acting mutagens was thought to be caused by a chemical reaction between MNU and the flavonoids. The half-lives of MNU in the presence or absence of isoliquiritigenin were compared, and the results were 21.0 ± 1.2 min and 20.0 ± 2.3 min, respectively. Furthermore, we could not detect any new product from a reaction mixture of MNU and isoliquiritigenin, and no significant change in the amount of isoliquiritigenin was observed by HPLC. These data indicated that isoliquiritigenin did not decompose MNU and did not scavenge on an electrophilic product generated from MNU in vitro.

**Fig. 5:**
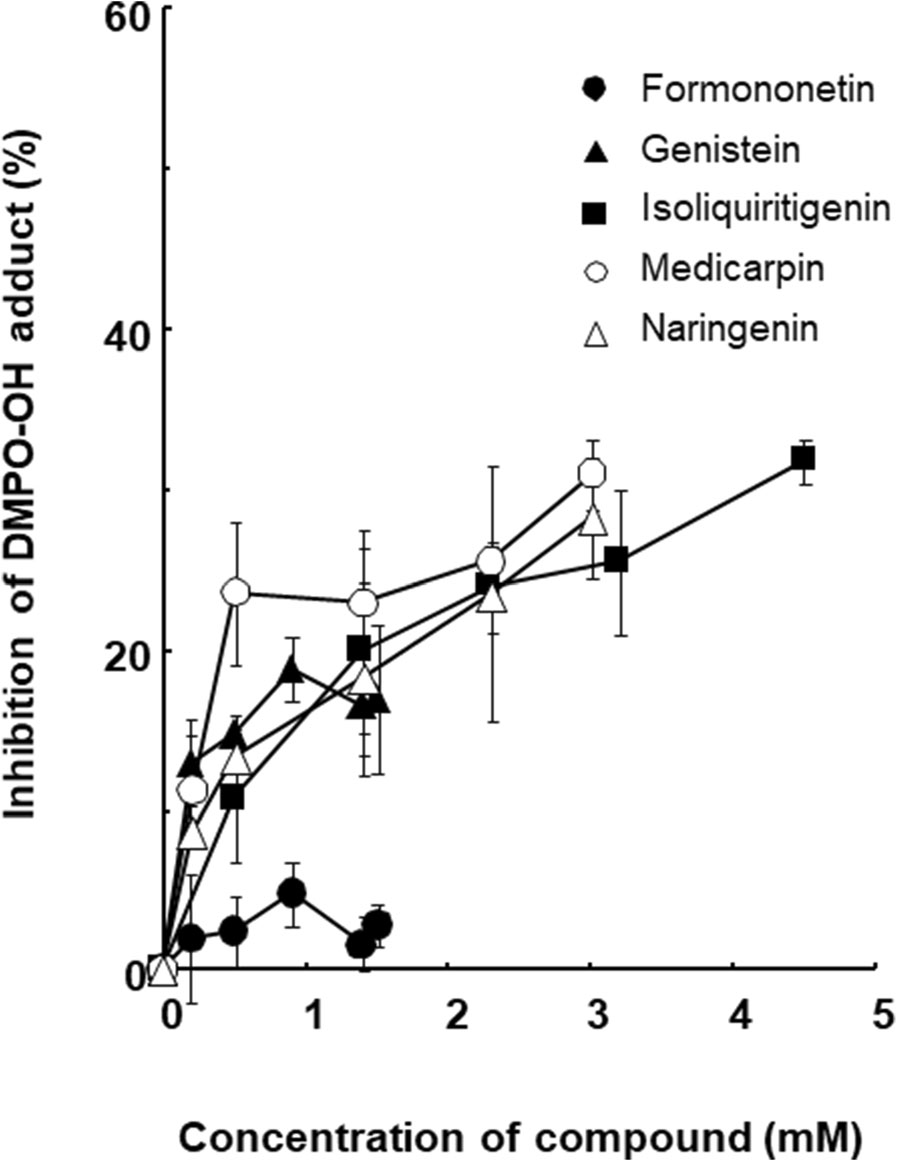
OH-scavenging activity of flavonoids

MNU treatments have been reported to induce not only DNA alkylation but also increase intracellular ROS levels [44, 45]. Therefore, five flavonoids were evaluated for their antioxidant activities using a hydroxyl radical (^•^OH)-scavenging assay (Fig. 5). To investigate the reaction of ^•^OH with flavonoids, the electron spin resonance (ESR) spin-trapping technique was used [46]. The Fenton reaction (Fe^2+^ + H_2_O_2_ → Fe^3+^ + ^−^OH + ^•^OH) was the source of ^•^OH [47], and 5,5-dimethyl-1-pyrroline *N*-oxide (DMPO) was used as the ^•^OH-trapping agent [48]. The capacity of the ^•^OH-scavenging activity is presented as the inhibition percent (%) relative to the intensity of the DMPO-OH adduct (Fig. 5).

Genistein, isoliquiritigenin, medicarpin and naringenin, which possess antimutagenic activity, inhibited the formation of the DMPO-OH adduct. Formononetin, which did not have antimutagenic activity towards MNU, showed very low ^•^OH-scavenging activities. These data indicated that antimutagenic activity against MNU-induced mutagenicity had the same tendency as the ^•^OH-scavenging activity.

Despite the fact that flavonoids scavenged ^•^OH, there were no significant changes in the amount of isoliquiritigenin in a reaction with MNU in a test tube. As a small amount of extracellular ROS was generated from MNU [44], the decrease of isoliquiritigenin was hard to detect.

Many studies have reported ^•^OH-scavenging activity of flavonoids [49] and the structure-activity relationship for free radical scavenging activity [50]. Furthermore, Makhafola et al. reported a direct correlation with antioxidant activity and antimutagenicity towards 4-nitroquinoline *N*-oxide [51]. Our results demonstrated that the hydroxyl radical scavenging activity of flavonoids was involved in their antimutagenicity against direct-acting MNU in the Ames assay with strain *S. typhimurium* TA1535. Since antimutagenicity of flavonoids toward MNU was significantly effective (approximately 100% inhibition), there are possibilities to have other antimutagenic mechanism. The flavonoids may block the reaction between methyldiazonium ion and DNA due to the interaction with DNA and flavonoids [52, 53]. Further investigation is required to quantify *O*^*6*^-methylguanine, which is mutagenic DNA adducts induced by MNU, in a reaction of flavonoids and DNA.

## Conclusions

It is important to prevent DNA damage by *N*-nitrosamines for cancer chemoprevention. In the present study, four components with antimutagenic activity against MNU from *S. suberectus* Dunn were identified as genistein, isoliquiritigenin, medicarpin, and naringenin. Isoliquiritigenin was the most active component of *S. suberectus* Dunn in inhibiting MNU-induced mutagenicity. This report describes the first demonstration of the antigenotoxic effects of these components against carcinogenic MNU.

Many flavonoids are known to have a bioactive effect on human health [54], and the dietary intake of flavonoids has been reported to reduce the risk of developing cancer, such as gastric, breast, prostate, and colorectal cancers [55]. The radical scavenging potency of flavonoids might be involved in their chemopreventive effects.

## Supplementary information

**Additional file 1: **Antimutagenic components in *Spatholobus suberectus* Dunn against *N*-methyl-*N*-nitrosourea.

## Data Availability

The datasets analysed during the current study are available from the corresponding author upon reasonable request.
