# Supplementary material for: Antimutagenic components in Spatholobus suberectus Dunn against N-methyl-N-nitrosourea
Source: Genes Environ. 2019 Dec 11;41:22. doi: 10.1186/s41021-019-0137-4 (PMC6907206; doi:10.1186/s41021-019-0137-4)
Supplement: Supplementary file 1 — Additional file 1: Antimutagenic components in Spatholobus suberectus Dunn against N-methyl-N-nitrosourea. [file 41021_2019_137_MOESM1_ESM.docx]

Antimutagenic components in *Spatholobus* *suberectus* Dunn against *N*-methyl-*N*-nitrosourea

Keiko Inami,^1,2^ Yoshihisa Asada,^2^ Takumi Harada^2,^ Yuta Okayama, ^1^ Noriko Usui^1^ and Masataka Mochizuki^1,2^

Contents

Effect of extracts of *Spatholobus* *suberectus Dunn* on MNU-induced mutagenicity in *S. typhimurium* TA1535 (Table S1) S1

Effect of extracts of methanol extract of *S. suberectus Dunn* on MNU-induced mutagenicity in *S. typhimurium* TA1535 (Table S2) S1

Effect of Fr.1–13 from ethyl acetate fraction on MNU-induced mutagenicity in *S. typhimurium* TA1535 (Figure S1) S2

Effect of Fr.2-1–2-8 on MNU-induced mutagenicity in *S. typhimurium* TA1535

(Figure S2) S2

Fractionation of Fr.2-4 (Figure S3) S3

Effect of Fr.3-1–3-9 on MNU-induced mutagenicity in *S. typhimurium* TA1535

(Figure S4) S4

Fractionation of Fr. 3-5-3 (Figure S5) S4

Effect of formononetin on MNU-induced mutagenicity in *S. typhimurium* TA1535

(Table S3) S5

Effect of genistein on MNU-induced mutagenicity in *S. typhimurium* TA1535

(Table S4) S5

Effect of isoliquiritigenin on MNU-induced mutagenicity in *S. typhimurium* TA1535

(Table S5) S6

Effect of medicarpin on MNU-induced mutagenicity in *S. typhimurium* TA1535

(Table S6) S6

Effect of naringenin on MNU-induced mutagenicity in *S. typhimurium* TA1535

(Table S7) S7

Reaction of MNU and isoliquiritigenin (Figure S6) S7

Inhibition (%) of DMPO-OH adducts of flavonoids (Table S8) S8

Table S1 Effect of extracts of *S. suberectus Dunn* on MNU-induced mutagenicity in *S. typhimurium* TA1535 (Data for Figure 1)

| mg/plate | Relative mutagenicity (%) | |
| --- | --- | --- |
|  | Methanol extract | Aqueous extract |
| 0 | 100 ± 0.0 | 100 ± 2.2 |
| 5 | 74 ± 12.0 | 88 ± 6.4 |
| 10 | 64 ± 14.7 | 89 ± 5.1 |
| 15 | 52 ± 14.8 | 86 ± 6.0 |
| 20 | 44 ± 11.5 | 81 ± 9.5 |

Table S2 Effect of extracts of methanol extracts of *S. suberectus Dunn* on MNU-induced mutagenicity in *S. typhimurium* TA1535 (Data for Figure 2)

| Extract | mg | Relative mutagenicity (%) |
| --- | --- | --- |
| Methanol | 7.5 | 77.9 |
|  | 15.0 | 66.9 |
| Ethyl acetate | 7.5 | 63.3 |
|  | 15.0 | 37.1 |
| *n*-Butanol | 7.5 | 84.2 |
|  | 15.0 | 81.1 |
| H_2_O | 7.5 | 93.3 |
|  | 15.0 | 87.9 |

**Isolation of antimutagenic compounds from ethyl acetate extract**

The ethyl acetate fraction was fractionated to No. 1–13 by silica gel column (Figure S1). Each of Fr.1–Fr.13 were prepared at concentration of 5.0 mg/50 μL DMSO, and the solutions were tested for mutagenicity induced by MNU (1.5 μmol/50 μL DMSO) (Figure S1).

Figure S1 Effect of Fr.1–13 fraction from the ethyl acetate fraction on MNU-induced mutagenicity in *S. typhimurium* TA1535

Fr.2 was fractionated to fractions 2-1–2-8 by ODS column (Figure S2). Each of Fr.2-1–Fr.2-8 were prepared at concentration of 0.5 mg/50 μL DMSO, and the solutions were tested for mutagenicity induced by MNU (1.5 μmol/50 μL DMSO) (Figure S2).

Figure S2 Effect of Fr.2-1–2-8 fraction from the ethyl acetate fraction on MNU-induced mutagenicity in *S. typhimurium* TA1535

Fr.2-4 showed the highest mutagenicity in *S. typhimurium* TA1535 among Fr.2-1–2-8 and then Fr.2-4 was fractionated as Figure S3. Finally, formononetin and medicarpin were identified from Fr.2-4-1-3-2 (Figure S3). The **structure** of formononetin [1] and medicarpin [2] were **confirmed** by **comparison** with previously reported spectral data (**^1^H-NMR and ^1^H-^1^H COSY**) from the **literatures.**

Figure S3 Fractionation of Fr.2-4

Fr.3 was fractionated to fractions 3-1–3-5 by HPLC. Each of Fr.3-1–Fr.3-9 were prepared at concentration of 5 mg/50 μL methanol, and the solutions were tested for mutagenicity induced by MNU (1.5 μmol/50 μL DMSO) (Figure S4).

Figure S4 Effect of Fr.3-1–3-9 fraction from the ethyl acetate fraction on MNU-induced mutagenicity in *S. typhimurium* TA1535

Fr.3-5 showed the highest mutagenicity in *S. typhimurium* TA1535 among Fr.3-1–3-9 and then Fr.3-5 was fractionated as Figure S5. Finally, isoliquiritigenin was identified from Fr.3-5-3-2 (Figure S5). The **structure** of isoliquiritigenin was **confirmed** by **comparison** with previously reported spectral data (**^1^H-NMR and ^1^H-^1^H COSY**) from the **literature [3].**

Figure S5 Fractionation of Fr.3-5

Table S3 Effect of formononetin on MNU-induced mutagenicity in *S. typhimurium* TA1535

(Data for Figure 4)

| Concentration | Revertants/plate±SE | Survival (%) | | MF (%) |
| --- | --- | --- | --- | --- |
| (µg/plate) |  | Colonies±SE | Survival rate (%) |  |
| 0 | 1385 ± 96 | 595 ± 6 | 100.0 | 100.0 |
| 50 | 1456 ± 76 | 577 ± 16 | 97.1 | 109.1 |
| 100 | 1379 ± 73 | 570 ± 17 | 95.8 | 104.6 |
| 250 | 1375 ± 102 | 583 ± 23 | 97.9 | 101.2 |
| 500 | 1461 ± 101 | 585 ± 14 | 98.3 | 107.5 |
| 1000 | 1439 ± 119 | 582 ± 17 | 97.7 | 106.1 |
| DMSO | 9 ± 1 | 605 ± 14 | 101.7 |  |

Table S4 Effect of genistein on MNU-induced mutagenicity in *S. typhimurium* TA1535

(Data for Figure 4)

| Concentration | Revertants/plate | Survival (%) | | MF |
| --- | --- | --- | --- | --- |
| (µg/plate) | ±SE | Colonies  ±SE | Survival rate (%) | (%) |
| 0 | 1670 ± 19 | 530 ± 10 | 100.0 | 100.0 |
| 50 | 1558　 ± 36 | 538 ± 11 | 101.8 | 92.3 |
| 100 | 1454 ± 28 | 546 ± 13 | 103.0 | 85.3 |
| 200 | 1307 ± 42 | 530 ± 3 | 100.3 | 79.8 |
| 300 | 1134 ± 59 | 535 ± 12 | 101.2 | 68.1 |
| 400 | 810 ± 23 | 530 ± 6 | 100.2 | 48.5 |
| 500 | 512 ± 15 | 537 ± 13 | 101.5 | 30.9 |
| DMSO | 7 ± 0 | 546 ± 10 | 103.3 |  |

Table S5 Effect of isoliquiritigenin on MNU-induced mutagenicity in *S. typhimurium* TA1535 (Data for Figure 4)

| Concentration | Revertants/plate±SE | Survival (%) | | MF (%) |
| --- | --- | --- | --- | --- |
| (µg/plate) |  | Colonies±SE | Survival rate (%) |  |
| 0 | 1729 ± 19 | 527 ± 9 | 100.0 | 100.0 |
| 100 | 1523 ± 8 | 534 ± 7 | 101.2 | 89.0 |
| 200 | 1230 ± 28 | 537 ± 11 | 101.8 | 72.5 |
| 300 | 651 ± 12 | 539 ± 8 | 102.2 | 37.6 |
| 400 | 393 ± 24 | 525 ± 11 | 99.6 | 22.4 |
| 500 | 160 ± 23 | 529 ± 5 | 100.5 | 8.9 |
| DMSO | 9 ± 1 | 536 ± 4 | 101.7 |  |

Table S6 Effect of medicarpin on MNU-induced mutagenicity in *S. typhimurium* TA1535

(Data for Figure 4)

| Concentration | Revertants/plate±SE | Survival (%) | | MF (%) |
| --- | --- | --- | --- | --- |
| (µg/plate) |  | Colonies±SE | Survival rate (%) |  |
| 0 | 2082 ± 60 | 596 ± 10 | 100.0 | 100.0 |
| 50 | 1873 ± 47 | 612 ± 10 | 102.7 | 87.7 |
| 100 | 1806 ± 35 | 615 ± 12 | 103.3 | 84.2 |
| 200 | 1559 ± 92 | 571 ± 13 | 95.8 | 77.8 |
| 300 | 1342 ± 62 | 590 ± 8 | 99.1 | 62.2 |
| 400 | 1183 ± 37 | 576 ± 14 | 96.5 | 58.7 |
| 450 | 930 ± 26 | 473 ± 13 | 79.4 | 56.0 |
| DMSO | 8 ± 0 | 573 ± 15 | 96.0 |  |

Table S7 Effect of naringenin on MNU-induced mutagenicity in *S. typhimurium* TA1535

(Data for Figure 4)

| Concentration | Revertants/plate±SE | Survival (%) | | MF (%) |
| --- | --- | --- | --- | --- |
| (µg/plate) |  | Colonies±SE | Survival rate (%) |  |
| 0 | 1632 ± 15 | 553 ± 20 | 100.0 | 100.0 |
| 50 | 1566 ± 30 | 569 ± 16 | 103.5 | 93.2 |
| 100 | 1331 ± 20 | 552 ± 17 | 100.5 | 81.7 |
| 250 | 1301 ± 34 | 550 ± 21 | 100.2 | 80.2 |
| 500 | 857 ± 31 | 563 ± 10 | 102.2 | 51.5 |
| 1000 | 205 ± 26 | 552 ± 9 | 100.4 | 12.1 |
| DMSO | 8 ± 1 | 588 ± 17 | 106.5 |  |

Figure S6 Reaction of MNU and isoliquiritigenin

1. Half-lives of MNU in the presence (●) or absence of isoliquiritigenin (〇)
2. Amount of [isoliquiritigenin] in the presence (●) or absence of MNU (〇)

Table S8 Inhibition (%) of DMPO-OH adducts of flavonoids (Data for Figure 5)

| Concentration  (mM) | Fomononetin | Genistein | Isoliquiritigenin | Medicarpin | Naringenin |
| --- | --- | --- | --- | --- | --- |
| 0 | 0.0±0.0 | 0.0±0.0 | 0.0±0.0 | 0.0±0.0 | 0.0±0.0 |
| 0.2 | 1.9±4.1 | 12.9±2.7 | － | 11.2±3.5 | 8.6±0.7 |
| 0.5 | 2.4±2.3 | 14.7±0.5 | 10.8±4.0 | 23.6±4.5 | 13.3±2.6 |
| 0.9 | 4.8±2.0 | 18.8±2.0 | － | － | － |
| 1.4 | 1.6±1.8 | 16.5±1.7 | 19.9±6.5 | 23.0±4.7 | 18.2±6.0 |
| 1.5 | 2.8±1.4 | 17.0±4.7 | － | － | － |
| 2.3 | － |  | 23.9±2.8 | 25.6±0.9 | 23.4±7.9 |
| 3.0 | － |  | － | 31.0±2.3 | 28.2±3.7 |
| 3.2 | － |  | 25.5±4.5 | － | － |
| 4.5 | － |  | 31.8±1.4 | － | － |

**References**

# Xiao HB, Krucker [M,](https://www.sciencedirect.com/science/article/pii/S0021967305000622#!) Putzbach K, Albert K. Capillary liquid chromatography–microcoil ^1^H nuclear magnetic resonance spectroscopy and liquid chromatography–ion trap mass spectrometry for on-line structure elucidation of isoflavones in Radix astragali. J Chromatogr A. 2005; 1067: 135–43.

1. Hasan [N](https://www.ncbi.nlm.nih.gov/pubmed/?term=Hasan%20N%5BAuthor%5D&cauthor=true&cauthor_uid=24280680), Osman [H](https://www.ncbi.nlm.nih.gov/pubmed/?term=Osman%20H%5BAuthor%5D&cauthor=true&cauthor_uid=24280680), Mohamad [S](https://www.ncbi.nlm.nih.gov/pubmed/?term=Mohamad%20S%5BAuthor%5D&cauthor=true&cauthor_uid=24280680), Wong [KC](https://www.ncbi.nlm.nih.gov/pubmed/?term=Wong%20KC%5BAuthor%5D&cauthor=true&cauthor_uid=24280680), Awang [K](https://www.ncbi.nlm.nih.gov/pubmed/?term=Awang%20K%5BAuthor%5D&cauthor=true&cauthor_uid=24280680), Zahariluddin ASM. The Chemical Components of Sesbania grandiflora Root and Their Antituberculosis Activity. Pharmaceuticals (Basel). 2012; 5(8): 882–9.
2. Namikoshi [M](https://www.jstage.jst.go.jp/search/global/_search/-char/en?item=8&word=MICHIO+NAMIKOSHI), Nakata [H](https://www.jstage.jst.go.jp/search/global/_search/-char/en?item=8&word=HIROYUKI+NAKATA), Nuno [M](https://www.jstage.jst.go.jp/search/global/_search/-char/en?item=8&word=MARIKO+NUNO), Ozawa [T](https://www.jstage.jst.go.jp/search/global/_search/-char/en?item=8&word=TAKASHI+OZAWA), Saitoh [T](https://www.jstage.jst.go.jp/search/global/_search/-char/en?item=8&word=TAMOTSU+SAITOH). Homoisoflavonoids and related compounds. III. Phenolic constituents of *Caesalpinia japonica* SIEB. *et* ZUCC. Chem Pharm Bull. 1987; 35: 3568–75.
